# The expression and clinical significance of SIRT5, H2A and TOP2A in cervical cancer

**DOI:** 10.3389/fonc.2026.1899320

**Published:** 2026-09-03

**Authors:** Fenghu Li, Xue Tian, Fan Mei, Yanjun Du, Lili Hu, Jiehui Li, Shengfa Su, Bing Lu

**Affiliations:** 1 Department of Oncology, The Affiliated Hospital of Guizhou Medical University, Guiyang, China; 2 Department of Oncology, School of Clinical Medicine, Guizhou Medical University, Guiyang, China; 3 Department of Gynecological Oncology, The Affiliated Cancer Hospital of Guizhou Medical University, Guiyang, China

**Keywords:** cervical cancer, chemoradiotherapy, H2A, SIRT5, survival, TOP2A

## Abstract

**Objective:**

To analyze the correlation between the expression of lysine deacetylase 5 (SIRT5), histone H2A and topoisomerase IIα (TOP2A) protein with clinical stage and survival in cervical cancer.

**Methods:**

The expression levels of SIRT5, H2A and TOP2A proteins were detected by immunohistochemical two-step method in paraffin-embedded tissue blocks from 65 patients with cervical cancer (21 cases of stage IB, 9 cases of stage IIA and 35 cases of stage IIIC1p). The expression distribution and positive rate of SIRT5, H2A and TOP2A proteins in tumor tissues of patients with different stages of cervical cancer were compared, and furthermore, survival analysis was conducted using Kaplan–Meier estimations and Cox regression modeling to pinpoint independent prognostic factors, along with the correlations among the expression levels of these three proteins.

**Results:**

The expression of SIRT5 and TOP2A increased significantly with the advancing clinical stage, whereas H2A expression showed a significant decrease with higher stage, and the difference was statistically significant (P < 0.05). As of April 2026, the median follow-up duration was 29 months (12–51 months). The 3-year overall survival (OS) and progression-free survival (PFS) of the SIRT5 negative group were significantly higher than those of the positive group (92% vs 49%, 90% vs 18%), and the differences were statistically significant (P < 0.05). In the H2A group, the 3-year OS and PFS of the positive group were significantly higher than those of the negative group (93% vs 64%, 86% vs 50%), and the differences were statistically significant (P < 0.05). The 3-year OS and FPS in the TOP2 A negative group were significantly higher than those in the positive group (87% vs 47%, 83% vs 19%), and the differences were statistically significant (P < 0.05). Multivariate Cox analysis identified SIRT5 positive expression (HR = 2.64, 95% CI: 1.08–5.60, P = 0.047) (HR = 2.24, 95% CI: 1.04–5.35, P = 0.004), TOP2A positive expression (HR = 2.96, 95% CI: 1.60–4.50, P = 0.018) (HR = 1.97, 95% CI: 1.29–4.13, P = 0.025) and H2A negative expression(HR = 0.29, 95% CI: 0.02–0.93, P = 0.032) (HR = 0.71, 95% CI: 0.08–0.98, P = 0.027) expression as independent predictors of OS and PFS. Spearman correlation analysis showed that the expression of SIRT5 protein was negatively correlated with the expression of H2A protein (r = -0.678, P = 0.000), and positively correlated with the expression of TOP2A (r = 0.652, P = 0.000).

**Conclusion:**

With the increase of clinical stage of cervical cancer, the positive expression rates of SIRT5 and TOP2A were significantly increased, and their high expression was related to the poor prognosis of patients. On the contrary, the negative expression rate of H2A increased with the advancing stage, and the prognosis of negative expression was worse. The above indicators had potential biomarker value for predicting the efficacy of cervical cancer treatment and the survival of patients. SIRT5 expression was negatively correlated with H2A and positively correlated with TOP2A. The potential regulatory mechanism among these three proteins warrant further in-depth investigation.

## 1 Introduction

Globally, approximately 661,000 new cases of cervical cancer are diagnosed annually, with about 80% of these cases occurring in undeveloped countries or regions (1, 2). As a developing country, China reports approximately 150,700 new cases of cervical cancer each year, with around 55,700 deaths (3). The common treatment methods of cervical cancer include surgery, radiotherapy and drug therapy. According to different International Federation of Gynecology and Obstetrics (FIGO) stages of cervical cancer, different treatment methods are adopted. For patients with early cervical cancer such as IA-IIA1, radical surgical resection can be performed. For locally advanced cervical cancer, platinum-based concurrent chemoradiotherapy is recommended, and immunotherapy can be added if necessary (4). However, approximately 30% of patients still experience recurrence and metastasis after standardized treatment, which affects their prognosis (5). In order to prevent recurrence and metastasis, it is usually recommended that patients regularly undergo pelvic and thoracoabdominal imaging examinations, as well as clinical indicators such as squamous cell antigen and CA125. However, the above indicators lack specificity. Therefore, exploring specific tumor markers for predicting the early recurrence and metastasis of cervical cancer has become a research hotspot. The lysine deacetylase (sirtuin) protein family is a class of nicotinamide adenine dinucleotide (NAD) -dependent deacetylases. As a subtype of the SIRT family and located in mitochondria, SIRT5 participates in the modification of proteins related to urea cycle, glycolysis, β-oxidation, or ketone body formation (6). The role of SIRT5 varies across different tumors. Research indicates that SIRT5 levels are elevated in primary liver cancer samples, and higher expression of SIRT5 in liver cancer tissues correlates with a reduced overall survival rate (7). High SIRT5 expression in breast cancer correlates with elevated metastasis risk and decreased one-year survival (8). Histone H2A variants exhibit distinct roles in gene transcription and DNA damage repair. Alterations in these variants are implicated in various conditions, including cancer, abnormal embryonic development, neurological disorders, metabolic diseases, and cardiovascular diseases (9). Topoisomerase IIα (TOP2A), predominantly localized in nuclear and mitochondrial compartments, serves as a critical regulatory factor for filament concentration and separation (10). Our previous research has established a strong association between TOP2A and tumor malignancy (11). Nevertheless, the literature has not yet addressed the involvement of SIRT5, H2A, and TOP2A in cervical cancer. This study aims to investigate the expression of these three markers in cervical cancer tissues and examine the potential correlation among their expressions. The findings may shed light on the impact of SIRT5 on the initiation, progression, and metastasis of cervical cancer through the regulation of H2A and TOP2A.The research protocol was approved by the Ethics Committee of the Affiliated Cancer Hospital of Guizhou Medical University (Approval No: FZ 2021-09-012).

## 2 Materials and methods

### 2.1 Inclusion criteria for cases were as follows

(1) Age range between 18 and 70 years; (2) Absence of preoperative treatments like radiotherapy or chemotherapy; (3) Pathologically confirmed as cervical squamous cell carcinoma, adenocarcinoma, or adenosquamous carcinoma; (4) Kanofsky score ≥70; (5) Availability of paraffin-embedded cervical cancer tissue specimens post-surgery; (6) Normal bone marrow hematopoietic function with white blood cell count >4.0×10^9/L, platelet count >100×10^9/L, and hemoglobin >70g/L; (7) Liver function within 1.5 times the upper limit of normal (ULN) for alanine aminotransferase, aspartate aminotransferase, and total bilirubin; renal function indicated by serum creatinine <1.5×ULN; (8) Absence of major organ dysfunction, hypertension, diabetes, coronary heart disease, or history of mental illness.

### 2.2 The relevant reagent models

SIRT5 (EPR23787-116), H2A (EPR17470) and TOP2A (TOP2A/1362) related antibodies were all purchased from ABCam Company. Dilute the antibody in accordance with the relevant reagent instructions. The specific ratios are as follows: SIRT5 (1:150), H2A (1:800), TOP2A (1:120). Add 150 μl of the anti-rabbit secondary antibody with horseradish peroxidase and incubate at room temperature for 15 minutes. Wash thrice with the cleaning solution, each time for two minutes.

### 2.3 Immunohistochemical staining

(1) Paraffin Tissue Sectioning: Secure the embedded wax block on the sectioning machine and cut sections with a thickness of 3 to 4 μm. Following sectioning, subject the sections to a baking process. (2) On-Machine Staining: Place the sections on the immunohistochemical staining instrument (model Leica BOND-III) and conduct staining according to the established protocol. This protocol includes dewaxing and hydration, inactivation of endogenous enzymes, antigen retrieval (The choice of acid or base for section repair treatment was determined by the primary antibody requirements. For instance, SIRT5 and H2A underwent incubation in EDTA antigen repair solution at pH 9.0 and 95 °C for 20 minutes, while TOP2A was treated in citric acid antigen repair solution at pH 6.0 and 95 °C for 25 minutes), incubation with primary antibodies, incubation with secondary antibodies, color development, and counterstaining. (3) Remove the sections and perform manual dehydration and transparent sealing. (4) Conduct microscopic examination and image analysis.

### 2.4 Interpretation of results adhered to the following standard

Examination of staining outcomes was conducted using the Olympus BX41TF microscope. Cells exhibiting brownish-yellow granules within the cytoplasm, cell membrane, or nucleus were deemed positive. SIRT5 predominantly localized in the cytoplasm and mitochondria, whereas H2A and TOP2A were primarily situated in the nucleus. Staining evaluation was independently conducted by two experienced pathologists. Staining intensity was scored from 0 to 3, corresponding to negative (0), weak (1), moderate (2), and strong (3). The proportion of positively stained tumor cells was graded as follows: 1 (≤25%), 2 (26%–50%), 3 (51%–75%), and 4 (>75%). The two scores were combined to calculate a composite IHC score, which was then categorized into negative (≤3), positive (>3).

### 2.5 Observation indicators

(1) Compare the expression distribution and positive expression rates of SIRT5, H2A, and TOP2A proteins in tumor tissues at different FIGO stages. (2) Examine the relationship between the expression and distribution of SIRT5, H2A, and TOP2A proteins and clinicopathological factors.

### 2.6 Statistical methods

Data analysis was conducted using SPSS version 20.0. Measurement data were presented as (mean ± standard deviation), and group comparisons were performed using the t-test. Count data were presented as n/% and analyzed using the chi-square test. The rank sum test was applied for comparing ordinal data. survival analysis was conducted using Kaplan–Meier estimations and Cox regression modeling to pinpoint independent prognostic factors. Spearman correlation analysis was utilized to examine the relationship between the protein expressions of SIRT5, H2A, and TOP2A in cancer tissues and clinical factors. Statistical significance was defined as P < 0.05.

## 3 Result

### 3.1 General patient information

A total of sixty-five patients with cervical cancer who underwent surgical resection at the Affiliated Cancer Hospital of Guizhou Medical University from January 2022 to April 2025 were analyzed. The median age of the cohort was 47 years (26 to 69 years). There were 21 cases classified as stage IB, 9 cases as stage IIA, and 35 cases as stage IIIC1p. Pathologically, 50 patients were diagnosed with squamous cell carcinoma, while 15 had adenocarcinoma. Postoperative adjuvant therapy was administered based on the presence of high-risk factors, such as lymph node metastasis, parametrial invasion, and positive resection margins, or the presence of intermediate-risk factors. Specifically, 3 patients received no treatment following surgery, 5 patients underwent radiotherapy alone, 26 patients were treated with concurrent chemoradiotherapy, and 31 patients received concurrent chemoradiotherapy in combination with adjuvant chemotherapy after surgery.

### 3.2 Expression of SIRT5, H2A, and TOP2A in cervical cancer at different stages

**Figure 1:**
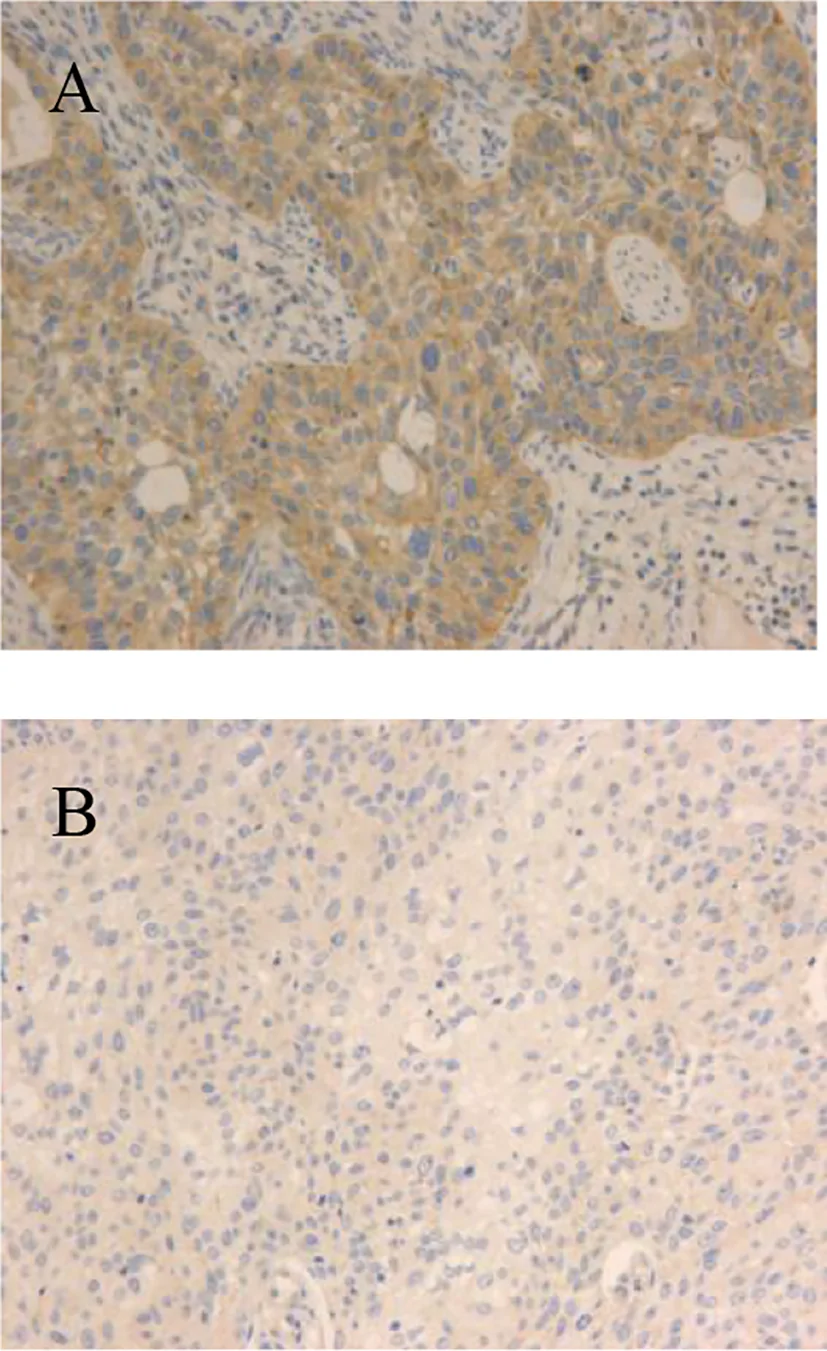
Immunohistochemical results of SIRT5 protein expression (x200) [**(A)** is positive, **(B)** is negative].

**Figure 2:**
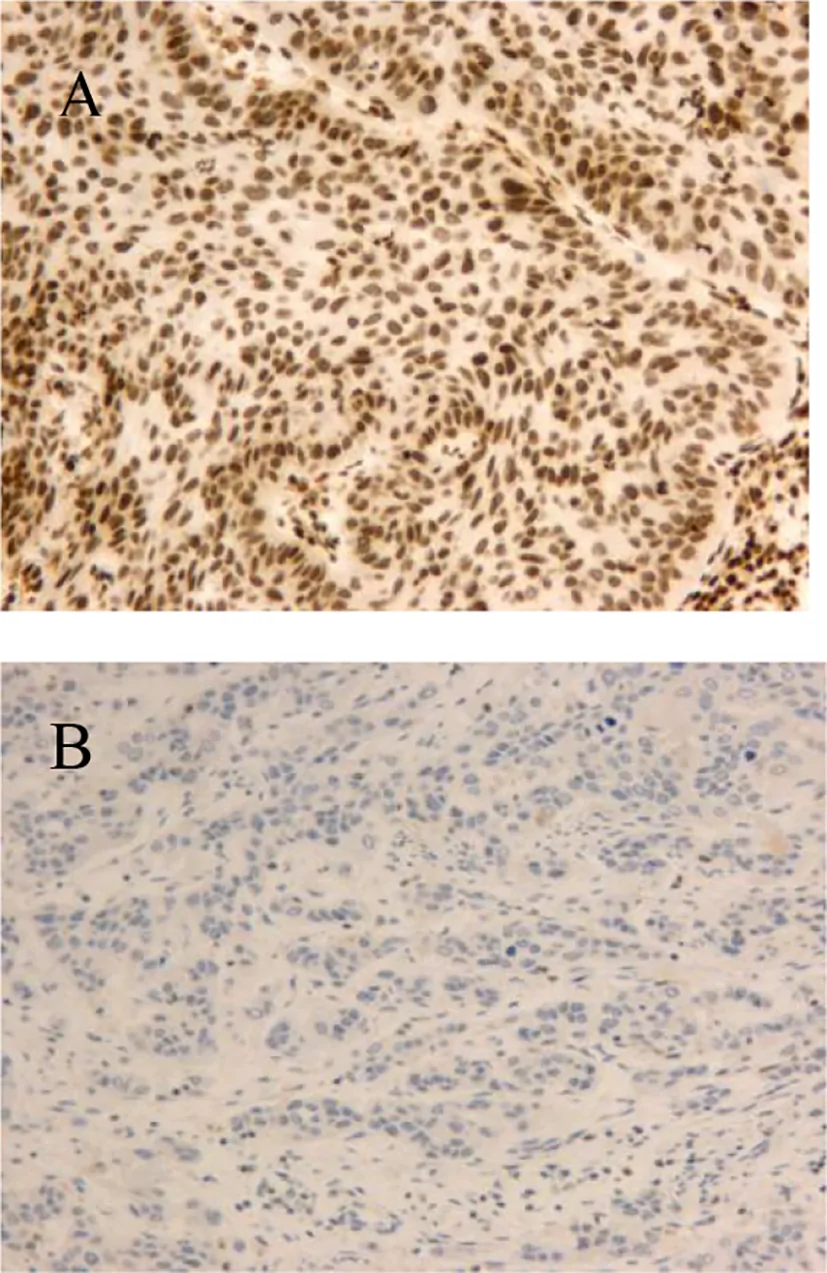
Immunohistochemical results of H2A protein expression (x200) [**(A)** is positive, **(B)** is negative].

**Figure 3:**
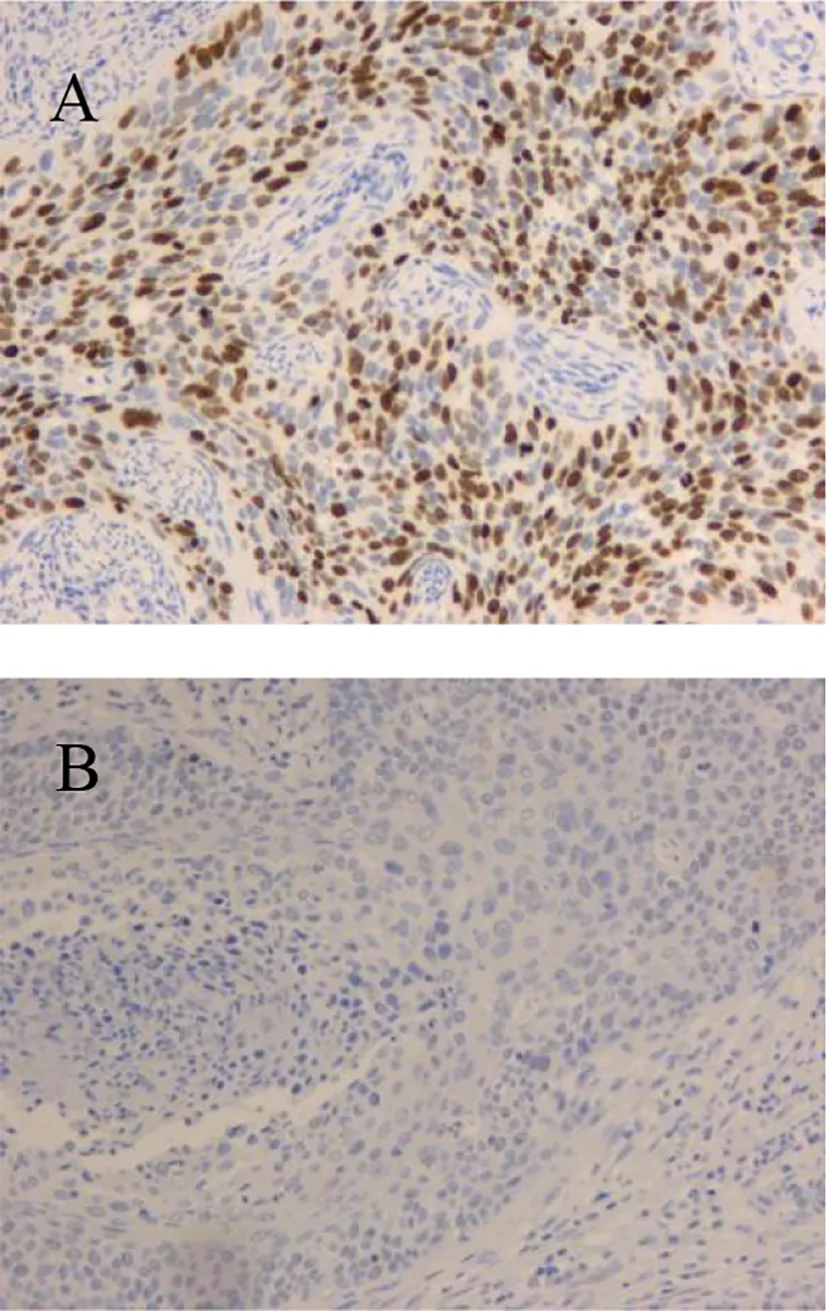
Immunohistochemical results of TOP2A protein expression (x200) [**(A)** is positive, **(B)** is negative].

The immunohistochemical results for the protein expression of SIRT5, H2A, and TOP2A were presented in **Figures 1**–**3**. The immunohistochemical scores revealed that the expression levels of SIRT5 and TOP2A significantly increased with advancing stage, whereas the expression of H2A exhibited a significant decrease as the stage progressed. These differences were statistically significant (P < 0.05), as outlined in **Table 1**.

**Table 1:** Expression and distribution of SIRT5, H2A and TOP2A in cervical cancer at different stages.

Parameters		IB	IIA	IIIC1p	t	P
SIRT5	positive	0 (0)	1 (11.1%)	19 (54.3%)	20.053	0.000
	negative	21 (100%)	8 (88.9%)	16 (45.7%)		
H2A	positive	16 (76.2%)	6 (66.7%)	7 (20.0%)	20.259	0.000
	negative	5 (23.8%)	3 (33.3%)	28 (80.0%)		
TOP2A	positive	0 (0%)	1 (11.1%)	11 (31.4%)	14.095	0.007
	negative	21 (100%)	8 (88.9%)	24 (68.6%)		

### 3.3 The correlation between SIRT5, H2A, and TOP2A and survival

**Figure 4:**
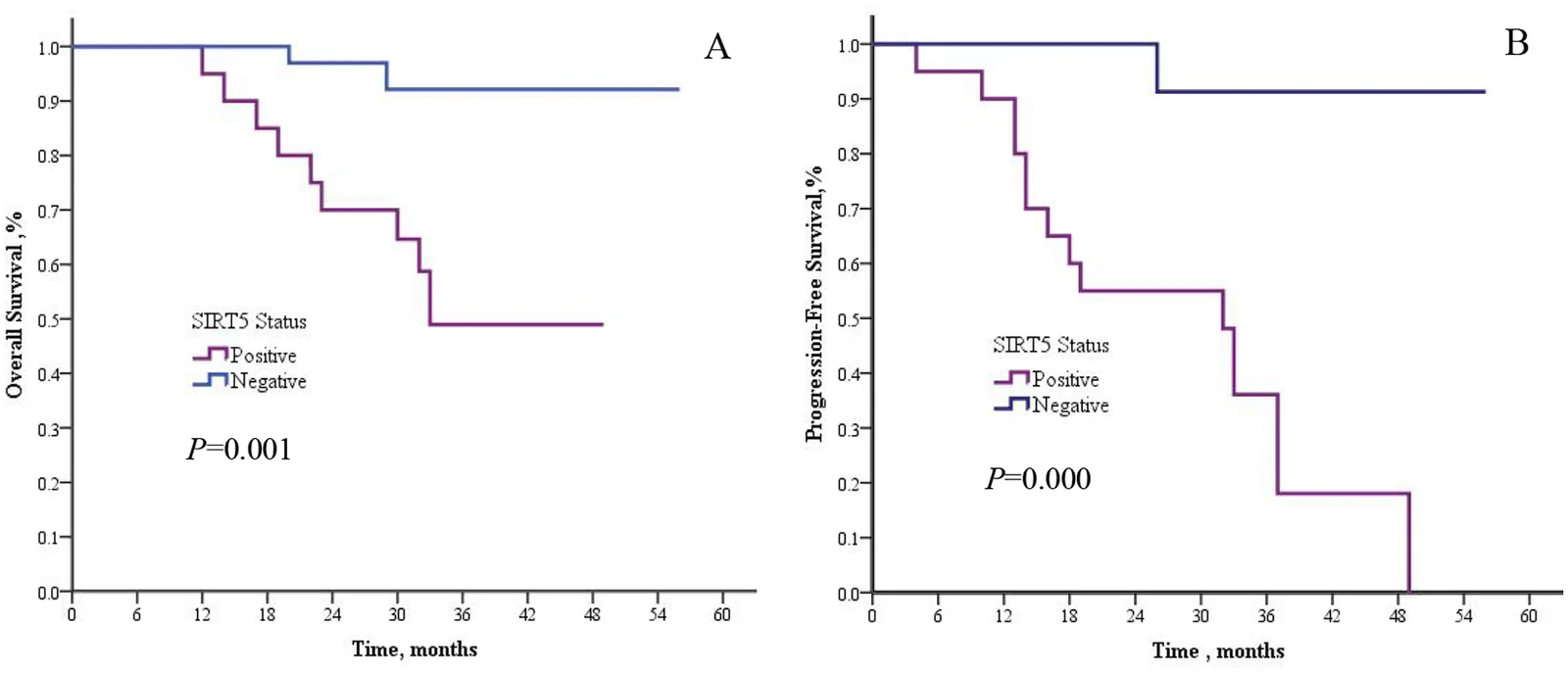
The relationship between different expression states of SIRT5 and survival [**(A)** represents OS, **(B)** represents PFS].

**Figure 5:**
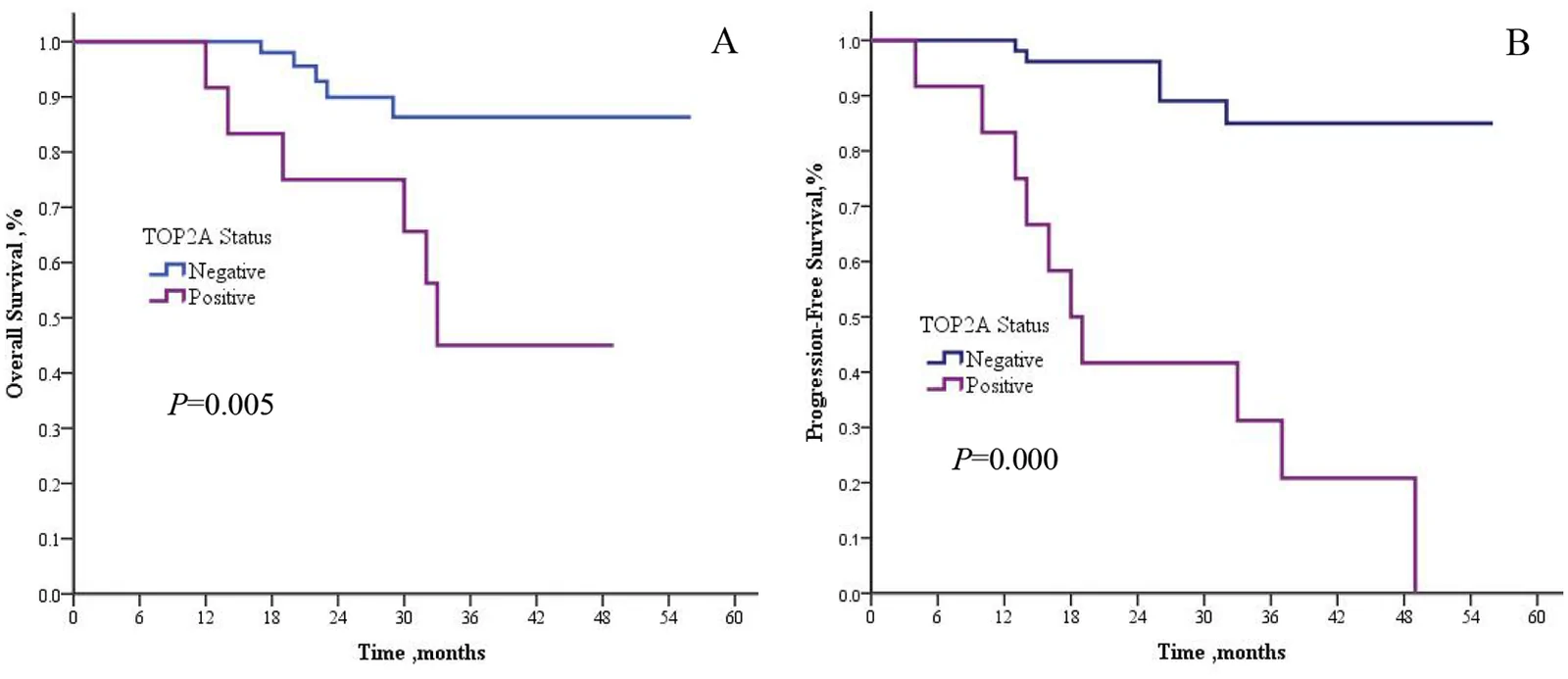
The relationship between different expression states of H2A and survival [**(A)** represents OS, **(B)** represents PFS].

**Figure 6:**
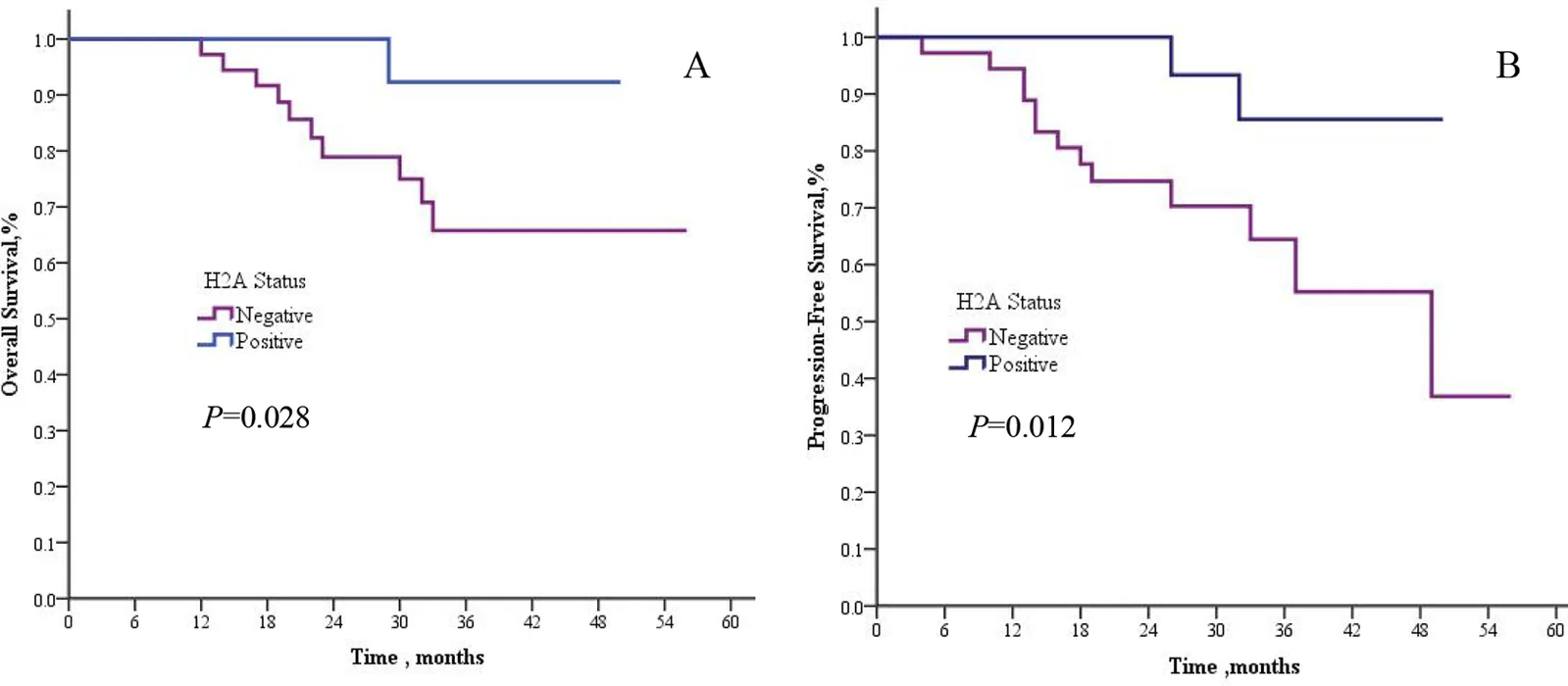
The relationship between different expression states of TOP2A and survival [**(A)** represents OS, **(B)** represents PFS].

To determine which clinicopathological features independently influenced OS and PFS, we applied multivariate Cox regression analysis. The evaluation included variables such as age, pathological type, tumor size, FIGO stage, lymph node metastasis, and expression of SIRT5, TOP2A and H2A. Univariate Cox analysis identified pathological type (P = 0.053, 0.048), tumor size (P = 0.033,0.036), FIGO stage (P = 0.021,0.016), lymph node metastasis (P = 0.038,0.026), and expression of SIRT5 (P = 0.005,0.000), TOP2A (P = 0.011,0.000) and H2A (P = 0.041,0.025) as variables that were significantly related to OS and PFS (**Table 2**). In multivariate analysis identified SIRT5 positive expression (HR = 2.64, 95% CI: 1.08–5.60, P = 0.047) (HR = 2.24, 95% CI: 1.04–5.35, P = 0.004), TOP2A positive expression (HR = 2.96, 95% CI: 1.60–4.50, P = 0.018) (HR = 1.97, 95% CI: 1.29–4.13, P = 0.025) and H2A negative expression (HR = 0.29, 95% CI: 0.02–0.93, P = 0.032) (HR = 0.71, 95% CI: 0.08–0.98, P = 0.027) expression as independent predictors of OS and PFS (**Table 3**). Patients were monitored until April 2026, with a median follow-up duration of 29 months (ranging from 12 to 51 months). The 3-year OS rate in the SIRT5-negative cohort was notably higher compared to the positive group (92% vs. 49%, P < 0.05). Likewise, the 3-year PFS rate in the SIRT5-negative cohort was significantly superior to the positive group (90% vs. 18%, P < 0.05), with all variances being statistically significant (refer to **Figure 4**). In the TOP2A category, the 3-year OS rate in the TOP2A-negative group was markedly higher than in the positive group (87% vs. 47%, P < 0.05). Similarly, the 3-year PFS rate in the TOP2A-negative group was significantly superior to the positive group (83% vs. 19%, P < 0.05), with statistically significant variances noted (refer to **Figure 5**). Regarding the H2A subgroup, the 3-year OS rate in the H2A-positive group was significantly greater than that in the negative group (93% vs. 64%, P < 0.05). Additionally, the 3-year PFS rate in the H2A-positive group was notably higher than in the negative group (86% vs. 50%, P < 0.05), with statistically significant differences observed (refer to **Figure 6**).

**Table 2:** Univariate Cox regression analysis for OS and PFS.

Characteristics	Hazard ratio (95%CI)	P value	Hazard ratio (95%CI)	P value
Age	0.91 (0.85-1.00)	0.163	0.94 (0.88-1.00)	0.151
Pathological type	1.50 (0.40-5.68)	0.053	1.51 (1.47-4.85)	0.048
Tumor size	1.14 (0.67-1.95)	0.033	1.43 (1.01-2.94)	0.036
FIGO stage	2.83 (1.99-4.37)	0.021	4.72 (1.34-16.57)	0.016
Lymph node metastasis	2.24 (1.05-4.11)	0.038	1.23 (1.07-3.84)	0.026
SIRT5	4.11 (2.02-8.51)	0.005	5.05 (2.01-10.23)	0.000
TOP2A	3.32 (1.04-7.51)	0.011	10.27 (3.50-30.18)	0.000
H2A	0.14 (0.02-0.79)	0.041	0.18 (0.04-0.81)	0.025
Therapeutic regimen	1.09 (0.82-1.47)	0.544	1.03 (0.79-1.33)	0.847

**Table 3:** Multivariate Cox regression analysis identifying independent prognostic factors of OS and PFS.

Characteristics	Hazard ratio (95%CI)	P value	Hazard ratio (95%CI)	P value
Pathological type	1.07 (0.23-3.64)	0.313	1.53 (0.41-4.23)	0.218
Tumor size	1.26 (0.71-2.26)	0.434	1.41 (0.85-2.35)	0.182
FIGO stage	3.61 (1.28-4.90)	0.016	2.25 (1.04-3.07)	0.047
Lymph node metastasis	2.88 (1.74-6.46)	0.020	1.53 (1.18-4.93)	0.037
SIRT5	2.64 (1.08-5.60)	0.047	2.24 (1.04-5.35)	0.004
TOP2A	2.96 (1.60-4.50)	0.018	1.97 (1.29-4.13)	0.025
H2A	0.29 (0.02-0.93)	0.032	0.71 (0.08-0.98)	0.027

### 3.4 The relationship between SIRT5 and the expression of H2A and TOP2A proteins was examined through immunohistochemical analysis

The results revealed a negative correlation between SIRT5 protein expression and H2A protein expression (r = -0.678, P = 0.000). Conversely, a positive correlation was observed between SIRT5 protein expression and TOP2A protein expression (r = 0.652, P = 0.000), as detailed in **Table 4**.

**Table 4:** Correlation between SIRT5 and H2A and TOP2A expression.

SIRT5	H2A			r	P	TOP2A			r	P
	+	-	Total			+	-	Total		
+	6	14	20			18	2	20		
–	42	3	45	-0.678	0.000	16	29	45	0.652	0.000
Total	48	17	65			34	31	65		

## 4 Discussion

The SIRT5 protein, situated in the mitochondrial ridge over a span of approximately 40.5 kDa, plays a crucial role in governing diverse biological processes encompassing metabolism, cell division, aging, and oxidative stress (12). Comprising ten exons, with eight being coding exons and two non-coding exons, SIRT5 functions as a binding site for Nicotinamide Adenine Dinucleotide (NAD+) (13). It features a sizable lysine acyl-binding pocket and exhibits desuccinylation and demalonylation activities (as desuccinase and demalonase), facilitating the preferential elimination of short-chain acyl groups like succinic acid, malonic acid, and glutaric acid from the lysine residues of various mitochondrial enzymes. This modification of proteins associated with the urea cycle, glycolysis, β-oxidation, or ketone body production contributes to its involvement in the initiation and progression of diverse tumors (14). Literature documents the varying impact of SIRT5 on tumor development. In liver cancer, reduced SIRT5 expression correlates with lower overall survival and higher recurrence rates (15). Conversely, high SIRT5 levels in lung cancer patients are linked to a notably shortened progression-free survival period (16). Notably, the relationship between SIRT5 expression and prognosis in cervical cancer remains unexplored in current literature. The findings of this study indicate that SIRT5 expression varies among cervical cancer patients at different stages, with lower expression observed in earlier stages. As the stage progresses, SIRT5 expression significantly increases (P < 0.05). Additionally, survival analysis demonstrated that SIRT5-positive patients exhibited significantly poorer outcomes compared to those in the negative group, both in terms of overall survival and progression-free survival (P < 0.05). Histone H2A, a pivotal histone in eukaryotes, undergoes diverse post-translational modifications that collectively form the histone code. This code modulates gene function by modifying chromatin structure or recruiting regulatory proteins, thereby propelling tumorigenesis. Anomalous H2A modifications are prevalent in tumors. *In vitro* investigations have demonstrated a correlation between heightened H2A levels post-radiotherapy and the radiosensitivity of 18 human cell types. Specifically, *in vitro* assays have shown an upsurge in H2A levels in radiotherapy-sensitive colorectal cancer cells under radiotherapy. The H2A variant, γH2A, is poised to emerge as a novel predictive marker for assessing the efficacy of preoperative radiotherapy in colorectal cancer patients (17). H2A is purported to possess anti-cancer attributes in gastrointestinal cancer, breast cancer, prostate cancer, head and neck cancer, and leukemia (9). Transcriptome analysis unveiled that ablation of H2A in breast cells results in elevated expression of epithelial-mesenchymal transition (EMT)-related genes. Subsequent experimental validation substantiated that downregulation of H2A in breast cancer cells leads to heightened activity of Twist1 and Slug transcription factors, orchestrating EMT and fostering tumor cell migration and invasion. Analogous to breast cancer, depletion of H2A.X induces EMT in colorectal adenocarcinoma cells (18). Post-translational modifications of H2A, including phosphorylation, succinylation, and malonylation, have been identified as critical factors in the pathophysiology of tumorigenesis. Consequently, the detection of H2A is considered a valuable approach for cancer prediction. Radiotherapy serves as the primary treatment modality for cervical cancer; however, there is a paucity of studies examining the relationship between H2A and the radiosensitivity of cervical cancer. In this study, all patients received local radiotherapy. The results indicated that the positive expression of H2A in early-stage patients was significantly greater than that in late-stage patients, and this expression correlated with survival outcomes. Notably, the survival rate of H2A-positive patients was significantly higher than that of H2A-negative patients (P < 0.05). These findings suggest that H2A positivity may serve as a predictor of radiosensitivity in cervical cancer. Research indicates that phosphorylation of H2A at site 120 can enhance its binding to TOP2A, thereby exerting mitotic effects on cells (19). Relevant studies (20) have demonstrated that the expression level of TOP2A in cervical cancer tissues is significantly elevated compared to normal tissues. However, correlation analyses between TOP2A and clinical stage, as well as survival outcomes, have been lacking. This study analyzed TOP2A expression across different stages of cervical cancer and found that TOP2A levels progressively increased with advancing stage. Notably, the expression of TOP2A in stage IIIC1p was significantly higher than in patients with stages I and II. Further survival analysis revealed that patients with positive TOP2A expression experienced significantly shorter overall survival and progression-free survival compared to TOP2A-negative patients (P < 0.05), suggesting that TOP2A may serve as a predictive indicator for clinical efficacy. As a distinct member of the SIRT family, SIRT5 possesses NAD+-dependent demalonylation activity, which is implicated in the modification of key glycolytic enzymes. This study also explored the relationship between H2A and SIRT5 in the context of demalonylation regulation. It was found that the expression of SIRT5 and H2A in tissues was negatively correlated, while both were positively correlated with the presence of the mitotic core regulatory enzyme TOP2A.

In conclusion, with the advancement of cervical cancer stages, there is a notable increase in the positive expression of SIRT5 and TOP2A, leading to poorer survival outcomes. Conversely, negative expression of H2A is associated with worsened survival as the disease progresses. These findings suggest the potential utility of these markers in predicting clinical outcomes. Moreover, SIRT5 exhibits a negative correlation with H2A expression but a positive correlation with TOP2A expression, indicating a possible interrelationship that warrants deeper investigation. Such insights could offer valuable evidence for identifying potential therapeutic targets in cervical cancer treatment.

## Data Availability

The original contributions presented in the study are included in the article/supplementary material. Further inquiries can be directed to the corresponding authors.
